# Acupuncture for Parkinson's disease-related constipation: current evidence and perspectives

**DOI:** 10.3389/fneur.2023.1253874

**Published:** 2023-08-31

**Authors:** Jiale Zhang, Xiaolei Ge, Kaiqi Zhang, Yun Qi, Shuo Ren, Xu Zhai

**Affiliations:** ^1^Institute of Basic Theory for Chinese Medicine, China Academy of Chinese Medical Sciences, Beijing, China; ^2^Wangjing Hospital, China Academy of Chinese Medical Sciences, Beijing, China; ^3^Department of Neurorehabilitation, Navy Qingdao Special Service Rehabilitation Center, Qingdao, Shandong, China; ^4^Department of Rehabilitation and Physiotherapy, Affiliated Hospital of Shandong University of Traditional Chinese Medicine, Jinan, China

**Keywords:** acupuncture, Parkinson's disease, constipation, mechanisms, randomized trials

## Abstract

Parkinson's disease-related constipation (PDC) is commonly associated with impaired dopamine transmission and gastrointestinal dysfunction. Current pharmacological treatments have limited efficacy and potential side effects. Acupuncture has shown promise as an alternative or adjunct therapy by modulating the brain–gut axis, gastrointestinal hormones, and autonomic function. Preliminary randomized trials have shown that acupuncture significantly improves constipation symptoms, bowel movements, and comfort compared to sham or drug treatments and is well-tolerated. The mechanisms of action may involve regulating the gut microbiota and mucosal immunity to improve dysbiosis and gastrointestinal motility. However, more rigorous studies are required to optimize acupuncture protocols and determine long-term efficacy and safety. In summary, acupuncture shows promise as an adjunct therapy for PDC, but further research is needed to confirm its efficacy and safety.

## Introduction

**Figure 1:**
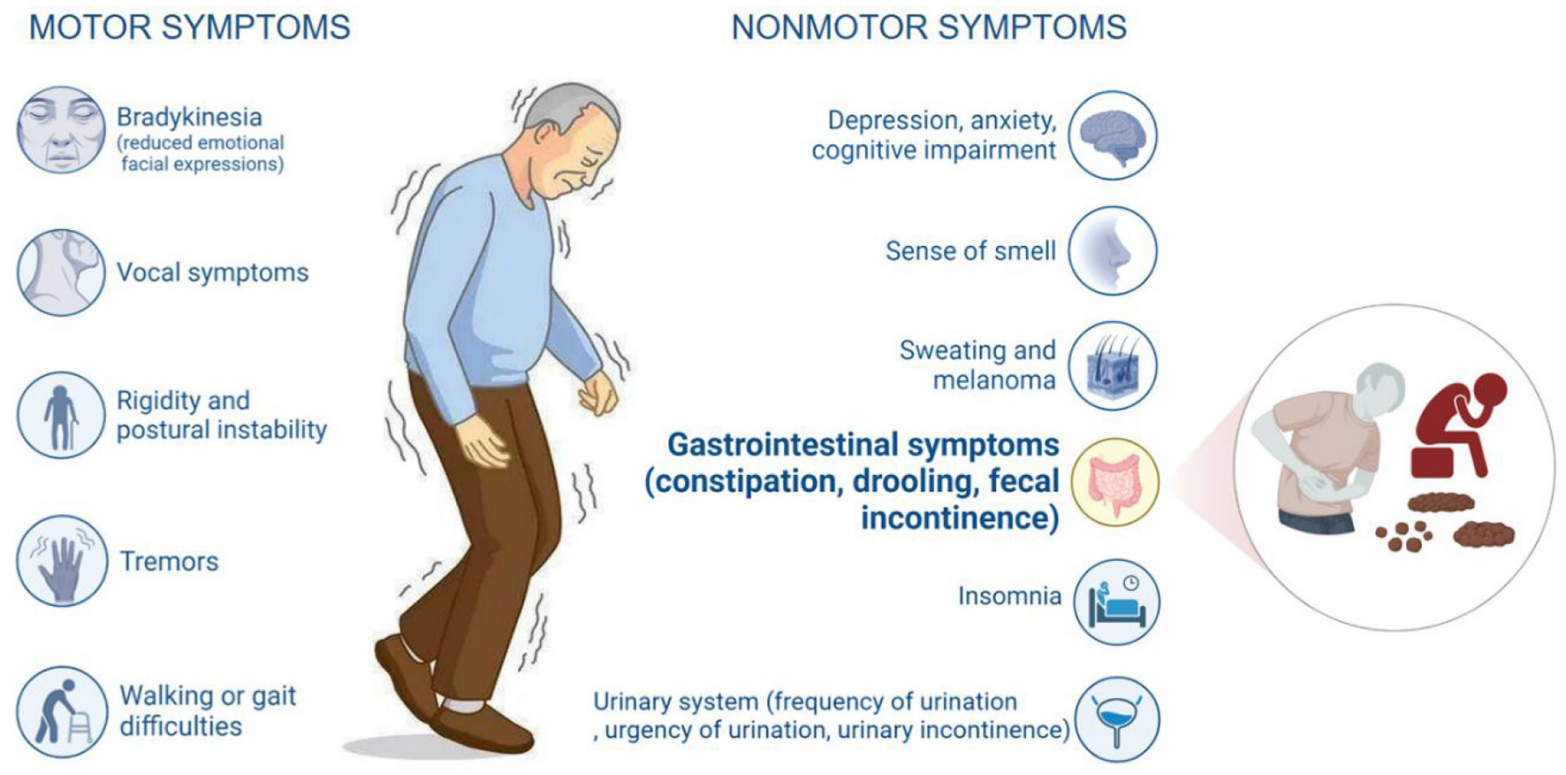
Symptoms of Parkinson's disease. The symptoms of Parkinson's disease can be categorized into motor and non-motor symptoms. Among the non-motor symptoms, gastrointestinal symptoms are an important category of clinical manifestations. They mainly include constipation, sialorrhea, and fecal incontinence.

Parkinson's disease (PD) is a common neurodegenerative disorder characterized by progressive loss of dopamine-secreting neurons in the substantia nigra, resulting in a reduction of dopamine and other neurotransmitters and subsequently the typical motor symptoms, including bradykinesia, tremors, muscle rigidity, and postural instability (1). In addition to motor symptoms, most PD patients suffer from gastrointestinal symptoms of varying severity, commonly including constipation, nausea, and dry mouth (2) (Figure 1). Parkinson's disease-related constipation (PDC) arises from impaired central nervous system control of bowel motility and digestive hormone secretion (3). It increases patients' physical burden and severely impacts their emotional wellbeing and quality of life.

The current clinical treatment of PDC focuses mainly on symptomatic relief. Primary treatments include laxatives to promote bowel peristalsis but can cause electrolyte imbalance with long-term use; fiber supplements to increase stool bulk and retain water but can cause bloating and pain if excessive; whole gut irrigation for refractory and fecal impaction constipation but also risks electrolyte disturbances and gut irritation (4). Moshapride shows some efficacy but is prone to dependency (5). Thus, safe and effective treatments that significantly alleviate symptoms are urgently needed. Acupuncture, a widely accepted traditional medical therapy through needling specific acupoints to regulate physiological and pathological states, has shown promise in improving bowel function in functional and post-stroke constipation (6, 7). Evidence from high-quality studies since 2023 also indicates that acupuncture produces evident clinical efficacy and safety in treating PDC (8, 9). Two randomized controlled trials (8, 9), in particular, found that acupuncture significantly increases bowel frequency, improves constipation and stool characteristics, relieves abdominal pain and discomfort, and enhances the quality of life compared with placebo or drug treatment. This perspective article timely summarizes recent research progress on PDC, focusing on advances in acupuncture treatment of PDC in recent years.

## Pathogenesis and treatments of Parkinson's disease-related constipation

PDC results from impaired central nervous system control of gastrointestinal motility and hormone secretion. It is closely related to the dysfunction of dopaminergic neurons. Dopamine acts through multiple receptors to regulate gastrointestinal activity (10). Reduced dopamine leads to weakened prokinetic effects mediated by D2 and 5-HT4 receptors, causing gastrointestinal motility disorders and delayed defecation reflex, resulting in constipation (11). Clinical manifestations of PDC (12, 13) include infrequent bowel movements, difficulty defecation, hard stools, and prolonged intervals between stools. Some patients also experience bloating and vomiting. The immobility in PD further aggravates constipation. Current treatments for PDC mainly include dopaminergic agents and 5-HT4 receptor agonists (14). However, their efficacy is limited, and dependency can develop with prolonged use. Motor symptoms also limit non-pharmacological treatments such as dietary adjustments and exercise. There is an urgent need for safe and effective therapies to improve constipation in PD patients. Important therapeutic strategies are novel approaches that can regulate gastrointestinal function without severe side effects.

## Mechanisms of acupuncture treatment

Acupuncture may affect gastrointestinal motility and hormone secretion by regulating the brain–gut axis and autonomic nervous function.

The brain–gut axis is a bidirectional communication network between the brain and gastrointestinal tract, mainly through the central nervous system (CNS), autonomic nervous system (ANS), and enteric nervous system (ENS) that influences gastrointestinal activity (15). The brain–gut axis involves brain regions such as the hypothalamus, hippocampus, amygdala, and spinal cord, connected to the gut *via* the vagus nerve and myelinated fibers (16). In PD, the linkage between the brain and the intestine can manifest in two distinct patterns (17): one is “brain-first,” which begins with α-synuclein deposition in the brain and subsequently spreads to the hypothalamus, medulla, spinal cord, and gastrointestinal tract, ultimately leading to the death of enteric neurons and gastrointestinal dysmotility. The other is “body-first,” which starts with abnormal gut microbiota or inflammation in the gastrointestinal tract, leading to enteric neuron death and α-synuclein deposition, ultimately affecting dopamine neurons, and the motor control center. Substance P is overexpressed in the olfactory neurons of PD patients possibly due to gut dysfunction and is relevant to the discussion of prodromal constipation (18). This supports the idea that the gut plays an important role in PD and that prodromal constipation may be an early sign of the disease. A study (19) suggests that prodromal constipation in *de novo* PD patients may serve as an important marker for disease progression as it can identify a subgroup with a distinct neurodegenerative trajectory, more severe motor impairment, and greater dopaminergic requirement in the mid-term. This highlights the close relationship between prodromal constipation and clinical presentation and prognosis of PD. This may help better understand PDC's pathophysiology and clinical presentations and provide guidance for developing more effective treatment and prevention strategies.

Acupuncture stimulation of specific brain areas and spinal cord segments can balance the activity of the sympathetic and parasympathetic nervous systems, thereby regulating the function of the brain–gut axis and improving gastrointestinal motility (20). By regulating autonomic mechanisms, electroacupuncture (EA) can improve gastrointestinal motility in normal and constipated rats (21). EA at ST36 (Zusanli) can increase fecal characteristics and accelerate gastric emptying, small intestinal transit, distal colon transit, and overall gastrointestinal transit times, and these effects are enhanced with prolonged EA treatment.

Acupuncture can promote gastrointestinal peristalsis and facilitate the expulsion of feces by increasing parasympathetic activity, thus improving constipation symptoms (22, 23). It can also balance the secretion of gastrointestinal hormones such as gastrin, gastric, and cholecystokinin, indirectly affecting gastrointestinal motility (24, 25). Recent animal studies (26) have demonstrated that EA at ST36 (Zusanli) in mice increased c-Fos expression in Barrington's nucleus of the pons, which projects to the sacral parasympathetic nucleus. This suggests Barrington's nucleus may mediate the effects of acupuncture on distal colonic motility, and these stimulatory effects occur *via* the sacral parasympathetic efferent pathway.

## Acupuncture modulates gut microbiota to improve constipation and gut-related disorders

The gut microbiota plays an important role in regulating gastrointestinal motility and homeostasis. Dysbiosis of the gut microbiota has been implicated in constipation. Acupuncture can improve gut dysbiosis and constipation by modulating gut microbiota and intestinal immunity. Studies have found that acupuncture can regulate the gut microbiota of slow transit constipation (27). It can upregulate probiotics such as *Lactobacillus* while downregulating pathogenic bacteria. This improves the diversity and balance of the microbiota.

The gut microbiota can regulate the expression of proteins and genes related to mucosal immunity in the gut, mediate the NF-κB signaling pathway involved in gut mucosal barrier protection, and maintain gut immune regulation and motility (28). In constipated patients, gut microbiota dysbiosis can lead to upregulated expression of serotonin transporter (SERT) in the intestinal epithelial cells, resulting in the absorption of excessive 5-HT and slowing of gut peristalsis (29). By transplanting feces from functional constipation patients into mice, researchers found that the mice had reduced colon motility, defecation frequency, stool quality, and water content as well as markedly elevated SERT expression in colonic Caco-2 cells and significantly decreased 5-HT levels (30).

Acupuncture can reduce the disease activity index in ulcerative colitis (UC) model rats and increase the abundance and diversity index of gut microbiota (31). Species identification showed that EA could increase the content of *Lactobacillus* and *Lachnospiraceae* bacterium while decreasing the content of *Clostridium bifermentans* in the gut microbiota of UC model rats (*P* < 0.05).

In stress gastric ulcer (SGU) model rats, the levels of TLR4 in brain and gut tissues were elevated, suggesting that gut microbiota dysbiosis may have activated the TLR4 signaling pathway, causing TLR4 overexpression (32). The increased TLR4 levels in the brain may also be related to its activation of the hypothalamic–pituitary–adrenal axis. After acupuncture, the levels of TLR4 in the brain and gut tissues of SGU rats were reduced, suggesting that acupuncture may inhibit immune responses mediated by TLR4, downregulate its overexpression, and thus improve gastric mucosal damage (21).

## Clinical research status of acupuncture for constipation associated with Parkinson's disease

To the best of our knowledge, research on acupuncture for PDC is still limited, with only a few studies published. We searched PubMed using the keywords “(acupuncture) AND (Parkinson)) AND (constipation).” While these studies employed randomized controlled trial (RCT) designs, their sample sizes were small and study quality needed improvement. Nonetheless, their initial results showed the potential therapeutic effects of acupuncture for alleviating clinical symptoms (Table 1).

**Table 1:** Summary of acupuncture studies for Parkinson's disease-related constipation.

**Journal**	**Intervention**	**Control**	**Treatment duration**	**Outcome measures**	**Acupoint**	**Evidence grade**
eClinicalMedicine	EA	Sham EA	12 weeks	UPDRS, PDQ-39, SBMs, VAS, CSS, PAC-QoL	ST25, SP14, RN2, GV21 to GB5, EX-HN1 to GB6, LI4, KI3, and LR3	Ib
Frontiers in Neuroscience	Acupuncture	Sham acupuncture	4 weeks	UPDRS, CSBMs, CSEAS, PAC-QoL	GV21, GV19, and next to GV20 1.5 cun bilateral, GV24, GV29, ST25, CV4, and ST37	Ib
Acupuncture in medicine	Acupuncture	NA	6 weeks	CSBMs, PAC-QoL	TE6, ST25, ST36, and ST37	3b

The table summarizes the key characteristics of the three acupuncture studies: journal, intervention, control, treatment duration, outcome measures, acupoints, and level of evidence. EA, electroacupuncture; UPDRS, Unified Parkinson's Disease Rating Scale; PDQ-39, 39-item Parkinson's Disease Questionnaire; SBMs, Spontaneous Bowel Movements; VAS, Visual Analog Scale; CSS, Constipation Severity Scale; PAC-QoL, Patient Assessment of Constipation Quality of Life; CSBMs, Complete Spontaneous Bowel Movements; CSEAS, Constipation Symptom and Efficacy Assessment Scale. The quality of the evidence level was evaluated according to the Oxford Centre for Evidence-based Medicine (CEBM) grading criteria.

### EA improves constipation and motor symptoms in PD: level Ib RCT

For instance, a study published in EClinicalMedicine (8) randomized 166 PD patients into electroacupuncture (EA) and waiting control groups. The EA group received 12 weeks of EA, while the control group received sham EA. Acupoints included GV21 (Qianding) and GB5 (Xuanlu) on both sides, EX-HN1 (Qianshencong) and GB6 (Xuanli) on both sides (two needles in each line), LI11 (Quchi), LI4 (Hegu), GB34 (Yanglingquan), ST36 (Zusanli), SP6 (Sanyinjiao), KI3 (Taixi), and LR3 (Taichong). For patients with constipation, additional acupoints were used: ST25 (Tianshu), SP14 (Fujie), and ST37 (Shangjuxu).

The primary outcome was the change in Unified Parkinson's Disease Rating Scale (UPDRS) score from the baseline to week 12. Secondary outcomes included motor and constipation disability evaluations. From the baseline to week 12, the 39-item Parkinson's Disease Questionnaire (PDQ-39) score decreased by 10 points in the EA group vs. 2.5 points in the control group. At weeks 16 and 24, the 20 m walk test showed a larger reduction from the baseline in the EA group. The EA group significantly decreased constipation severity, with a change in CCS of −2.3 (95% CI −4.3 to −0.3) at week 12, compared to the control group, with a change of −0.5 (95% CI −2.5 to 1.5). The EA also increased the number of spontaneous bowel movements per week, from a median of 2.5 (IQR 0.5–2.8) at the baseline to 3.0 (IQR 1.0–4.0) at week 12. In contrast, the control group had no change in bowel movements (median 2.0, IQR 0.0–3.0). These results suggest that combined drug and EA treatment can significantly increase bowel movements, with EA showing better effects. No adverse effects related to acupuncture were reported.

According to the Oxford Centre for Evidence-Based Medicine (CEBM), this is a level Ib RCT. Considering the limited evidence, EA could be recommended as part of integrated PD management though individual patient considerations should guide treatment choices. However, more high-quality studies are needed to validate these preliminary findings.

### Acupuncture improves constipation symptoms in PDC: level Ib RCT

Another study published in Frontiers in Neuroscience (9) evaluated the efficacy of acupuncture for PDC.

The study recruited 78 PD patients with constipation and randomly assigned them to acupuncture and sham acupuncture groups. The acupuncture group received true acupuncture treatment, while the sham group received simulated acupuncture. Both groups received 12 treatments over 4 weeks.

Acupoints included Sishenzhen: four acupoints, consisting of GV21 (Qianding), GV19 (Houding), and next to GV20 (Baihui) 1.5 cun bilateral, GV24 (Shenting), GV29 (Yintang), ST25 (Tianshu), CV4 (Guanyuan), and ST37 (Shangjuxu). All acupoints were treated bilaterally according to Chinese national standards (GB/T12346-2006).

Finally, 76 patients completed all treatments and follow-ups. Efficacy evaluations included the constipation scoring system (CSEAS), Patient Assessment of Constipation Quality of Life Questionnaire (PAC-QoL), and UPDRS. After treatment and during follow-up, the acupuncture group had significantly lower CSEAS total scores compared to the sham group, with differences of −4.77 (95% CI, −5.97 to −3.57; *P* < 0.001) after treatment and −4.00 (95% CI, −5.27 to −2.74; *P* < 0.001) during follow-up. Additionally, acupuncture improved difficulty, Bristol stool form, and time spent for straining more than sham acupuncture. These results suggest acupuncture can significantly improve constipation symptoms in PDC patients and enhance their quality of life. No serious adverse events were reported, and the self-limiting nature of mild adverse events reported in the acupuncture group, such as hematoma, ecchymosis, dizziness, and abdominal pain.

According to the CEBM level grading system, this article can be graded as level Ib evidence (single high-quality RCT within a systematic review).

### Acupuncture improves bowel movements in PD: evidence from a case report

Furthermore, case reports highlight the potential benefits of acupuncture for Parkinson's disease-related constipation. One case report (33) described a 75-year-old PD patient who received acupuncture at TE6 (Zhigou), ST25 (Tianshu), ST36 (Zusanli), and ST37 (Shangjuxu) for 18 treatments over 6 weeks.

Outcomes included weekly complete spontaneous bowel movements (CSBMs) and the PAC-QoL, assessed before treatment (1–2 weeks pre-treatment) and during treatment at 6 weeks. CSBMs increased weekly during treatment and PAC-QoL scores decreased. No adverse events were observed during treatment. The therapeutic effect persisted at 1 month follow-up. The results showed that acupuncture effectively increased bowel movements and improved constipation-related quality of life in PD patients with constipation.

The level of evidence of this study is 3b.

Currently, the total sample size of acupuncture studies for PD constipation is only over 240 cases. Although existing studies have small sample sizes, preliminary results show that acupuncture is more effective in improving symptoms, increasing bowel movements and relieving discomfort. This suggests that acupuncture may relieve PDC symptoms and improve patient quality of life through multiple mechanisms, such as balancing autonomic function, increasing gastrointestinal hormone secretion, and regulating the brain–gut axis.

## Acupuncture for PDC: perspectives

Acupuncture is a promising therapeutic approach for PDC that has shown potential in regulating gut dysbiosis, improving intestinal immunity, and enhancing gut motility. While the evidence supporting acupuncture for PDC is limited, the findings thus far are encouraging. With further research, acupuncture may become a valuable addition to the current treatment options for PDC. Numerous studies have demonstrated that acupuncture elicits the release of neurotransmitters such as the substance P and serotonin, which are involved in gut–brain axis signaling (34, 35). Despite insufficient evidence, acupoints stimulating the head and face might function *via* the “brain-first” model. In contrast, acupoints associated with the gastrointestinal tract might adhere to the “body-first” model. However, further investigation is imperative to comprehensively elucidate how acupuncture impacts the gut–brain axis, understand neurotransmitter changes, explore inflammatory responses, and more. By delving into these aspects, we can gain a deeper understanding of the therapeutic effects of acupuncture and its potential application in PDC.

In addition to investigating the mechanisms, addressing the methodological considerations in clinical research is crucial. Conducting large-scale randomized controlled trials will provide more robust evidence regarding the efficacy of acupuncture for PDC. Furthermore, improving efficacy assessment methods and comparing acupuncture with other treatment modalities will help establish its comparative effectiveness and guide clinical decision-making. These requirements in clinical research are vital steps toward validating acupuncture as a viable treatment option for PDC.

Future studies should also focus on optimizing acupuncture treatment protocols tailored explicitly for PDC. Refining acupuncture sessions' frequency, duration, and intensity can maximize their therapeutic outcomes in PDC management. Additionally, long-term studies are needed to evaluate the sustained effects of acupuncture on PDC symptoms, providing insights into its long-term efficacy and durability as a treatment option. Furthermore, exploring the potential synergistic effects of combining acupuncture with other therapies, such as medications and lifestyle modifications, holds promise in enhancing treatment outcomes for individuals with PDC. This integrative approach can address the multifaceted nature of PDC, targeting underlying pathophysiological mechanisms while managing symptoms. However, it is essential to exercise caution when recommending acupuncture, given the limited real-life evidence. Rigorous clinical trials and real-world studies are necessary to establish the safety and effectiveness of acupuncture as adjunctive therapy for PDC.

In summary, by addressing the gaps in current research and charting future research directions, we can further enhance our understanding of the prospects of acupuncture for Parkinson's disease-related constipation. Investigating the mechanisms, conducting robust clinical trials, optimizing treatment protocols, and exploring combination therapies will contribute to building a comprehensive body of evidence. This will ultimately pave the way for acupuncture to emerge as a mainstream therapeutic option, providing practical and holistic approaches to managing Parkinson's disease-related constipation.

## Data availability statement

The original contributions presented in the study are included in the article/supplementary material, further inquiries can be directed to the corresponding authors.

## Author contributions

JZ: Writing—original draft, Writing—review and editing. XG: Writing—original draft, Writing—review and editing. KZ: Formal analysis, Resources, Writing—review and editing. YQ: Formal analysis, Writing—review and editing. SR: Conceptualization, Writing—review and editing. XZ: Conceptualization, Writing—review and editing.
